# Supplementary material for: HIVprotI: an integrated web based platform for prediction and design of HIV proteins inhibitors
Source: J Cheminform. 2018 Mar 9;10:12. doi: 10.1186/s13321-018-0266-y (PMC5845081; doi:10.1186/s13321-018-0266-y)
Supplement: Supplementary file 2 — Additional file 2. Source code of HIVProtI web server. [file 13321_2018_266_MOESM2_ESM.zip › HIVprotI_Source-code/hivprotI/weka-3-6-6/doc/weka/gui/graphvisualizer/LayoutCompleteEvent.html]

LayoutCompleteEvent


---


|  |  |  |
| --- | --- | --- |
| **Overview** \| **Package** \| **Class** \| **Tree** \| **Deprecated** \| **Index** \| **Help** \| **Weka's home** | |  |
| **PREV CLASS**   **NEXT CLASS** | **FRAMES**    **NO FRAMES**     **All Classes** |  |
| SUMMARY: NESTED \| FIELD \| CONSTR \| METHOD | DETAIL: FIELD \| CONSTR \| METHOD |  |


---


## weka.gui.graphvisualizer Class LayoutCompleteEvent

```
java.lang.Object
  java.util.EventObject
      weka.gui.graphvisualizer.LayoutCompleteEvent
```

**All Implemented Interfaces:**: java.io.Serializable

---

``` public class LayoutCompleteEvent extends java.util.EventObject ```

This is an event which is fired by a LayoutEngine once
a LayoutEngine finishes laying out the graph, so
that the Visualizer can repaint the screen to show
the changes.

**Version:**
:   $Revision: 1.5 $ - 24 Apr 2003 - Initial version (Ashraf M. Kibriya)

**Author:**
:   Ashraf M. Kibriya (amk14@cs.waikato.ac.nz)

**See Also:**: Serialized Form

---

**Constructor Summary**

`LayoutCompleteEvent(java.lang.Object source)`


**Method Summary**

**Methods inherited from class java.util.EventObject**

`getSource, toString`

**Methods inherited from class java.lang.Object**

`equals, getClass, hashCode, notify, notifyAll, wait, wait, wait`

**Constructor Detail**

### LayoutCompleteEvent

```
public LayoutCompleteEvent(java.lang.Object source)
```


---


|  |  |  |
| --- | --- | --- |
| **Overview** \| **Package** \| **Class** \| **Tree** \| **Deprecated** \| **Index** \| **Help** \| **Weka's home** | |  |
| **PREV CLASS**   **NEXT CLASS** | **FRAMES**    **NO FRAMES**     **All Classes** |  |
| SUMMARY: NESTED \| FIELD \| CONSTR \| METHOD | DETAIL: FIELD \| CONSTR \| METHOD |  |


---
